# The Synergism of Platinum-Gold Bimetallic Nanoconjugates Enhances 5-Fluorouracil Delivery In Vitro

**DOI:** 10.3390/pharmaceutics11090439

**Published:** 2019-09-01

**Authors:** Vareessh Maney, Moganavelli Singh

**Affiliations:** Nano-Gene and Drug Delivery Group, Discipline of Biochemistry, University of KwaZulu-Natal, Private Bag X54001, Durban, South Africa

**Keywords:** 5-fluorouracil, bimetallic nanoparticles, cancer, gold, platinum, pH-responsive

## Abstract

Nanoparticle application has significantly impacted the field of medicine. The need to develop novel drugs with higher therapeutic potential has stimulated the development of innovative delivery strategies to mitigate the potent side effects associated with known chemotherapeutic drugs. This paper describes the synthesis of platinum-gold bimetallic nanoparticles (PtAuBNps), their functionalisation with chitosan, and entrapment of the anticancer drug 5-fluorouracil (5-FU). All PtAuBNps and their drug nanocomposites were physico-chemically characterised, displaying desirable properties with regards to shape, size (<120 nm) and colloidal stability. 5-FU binding and loading capacities in PtAuBNps were found to be 90.17% and 22.56%, respectively. In vitro cytotoxicity profiles determined using the MTT and SRB assays reflected up to 65% cell death in the MCF-7, HepG2 and Caco-2 cell lines. These nanocomposites exhibited excellent physiochemical attributes, high specificity towards cancer cells, with a pH-sensitive drug release in a simulated acidic tumour microenvironment through zero-order release kinetics. In addition, they possessed the potential to traverse the mucosal lining facilitating oral drug administration. Overall, 5-FU encapsulation improved the bioavailability of the drug in cancer cells, with the promise of enhancing its therapeutic effect, biocompatibility and safety. These positive results highlight PtAuBNps as promising in vitro delivery systems and merits future in vivo research.

## 1. Introduction

Cancer, the unrestrained proliferation of dysfunctional cells, is a leading cause of mortality worldwide. Several efforts have been made to advance treatment regimens. However, the astounding complexity at cellular, genetic and epigenetic levels allow this menacing disease to show great diversity, to resist treatment, re-emerge and invade surrounding tissue. Some of the well-established treatment options include surgery, radiation, hormonal therapy, immunotherapy and hyperthermia [1]. Chemotherapy utilises therapeutic compounds either alone or in combination to thwart cellular proliferation in both solid tumours and haematological cancers. 

5-FU is a hydrophilic, water soluble, antimetabolite drug that is used extensively in clinical chemotherapy for the treatment of breast, brain, liver, pancreatic and lung cancers [2]. It is often a stand-alone drug for treatment of colorectal cancers, and together with modulators such as leucovorin and oxaliplatin, is the most effective treatment for metastatically advanced colorectal cancers [3]. However, its clinical application has limitations, including dose dependent side effects, rapid metabolism in vivo, short half-life, non-uniform oral absorption, compromised tissue penetration and non-selective biodistribution [4]. To overcome these underlying predicaments, the incorporation of potent biological agents together with metal nanoparticle carriers is rapidly gaining momentum. The desirable inherent properties of nanomaterials hold great promise in the treatment of cancer. Their favourable size, shape and surface morphology are bringing the “magic bullet” concept envisioned by Paul Ehrlich into realisation. Nanotechnology provides a dynamic strategy to exploit pathophysiological tumour abnormalities, bypass tedious biological barriers, infiltrate deep into subcellular compartments, and deliver therapeutic agents to their pathological target site, thus improving their therapeutic efficiency [5,6].

Of the multitude of framework materials envisioned to deliver drugs safely, AuNps and PtNps are promising, owing to their inert core, high atomic number and enhanced optical and structural properties. Moreover, they can be easily fabricated within a favourable biomedical size range to possess high colloidal stability giving them the advantage over other nanoparticulate systems [7]. Noble metals also possess the ability to absorb light or radio waves, which has generated novel non-invasive cancer therapy options including, photodynamic therapy (PDT) and radiotherapy, which can be utilised with chemotherapy for better eradication of tumour cells [8]. While AuNps are bio-inert, stable and relatively non-toxic in biological systems, PtNps possess chemotherapeutic and chemo-preventative properties [9,10]. These remarkable features have inspired research into the synthesis of hybrid PtAuBNps, imbued with properties of both metals, as well as novel properties through quantum confinement and synergism. The synergistic combination of noble metals has exhibited potential in nanomedicine, particularly in cancer therapy as drug delivery vehicles and theranostic agents [11]. Modern metallurgy on the nanoscale present unique features, including an enhanced band gap, surface chemistry, photoluminescence, electrical and magnetic properties that rival monometallic systems [12]. Furthermore, by embracing surface functionalisation paradigms, these modern platforms can be tailored to release their payloads through either passive and sustained drug release or active targeting for site-specific drug delivery.

Chitosan (CS), a polycationic biopolymer is an exceptionally popular stabilising agent in drug delivery, owing to its biodegradability, non-toxicity, mucoadhesiveness, feasibility and permeation enhancing effects [13]. In recent years, CSNps have emerged as promising carriers for sustained release preparations, to improve storage stability, solubility and prolong the half-life of anticancer drugs [14]. The ionic gelation reaction based on the electrostatic interaction between the amine group of chitosan and TPP is a facile and inexpensive way to form chitosan nanospheres endowed with its inherent characteristics [15]. The entangled polymeric framework provides a large surface area to volume ratio for the encapsulation of PtAuBNps and 5-FU. In addition, the flexible nature of the cross-linked framework allows the tailored release of drugs through pH-induced gel-sol transitioning [16,17]. The addition of the PEG bearing emulsifier Tween 80 stabilises and confers the CS nanocomposites with fusogenic properties similar to dioleoylphosphatidylethanolamine (DOPE) [18]. This is essential for successful endolysosomal escape, and maximising the transport of 5-FU into the nucleus. It is anticipated that precisely engineered target activated delivery systems, instilled with good physiochemical characteristics, will bring about site-specific cancer targeting through apoptosis induction. 

**Figure 1:**
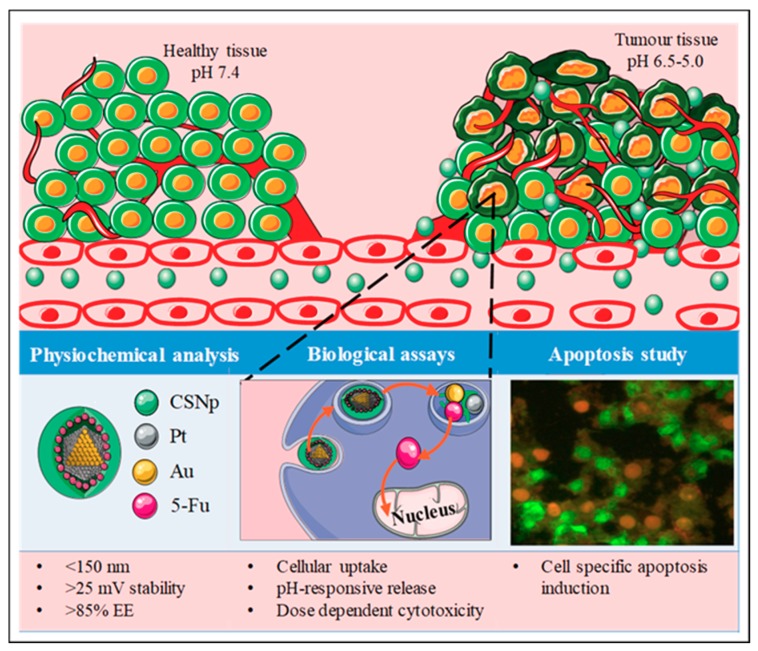
Schematic illustration of 5-fluorouracil encapsulated platinum-gold/chitosan bimetallic nanoparticles for systemic delivery and release to cancer cells leading to cytotoxic responses and apoptosis induction.

In this study, PtAuBNps and 5-FU were enclosed in an entangled CS framework to support: (1) favourable physiochemistry; (2) high mucoadhesive propensity; (3) pH-responsive release; and (4) site-specific induced toxicity in vitro (Figure 1). Currently, there is a dearth of scientific knowledge regarding the cytotoxicity, delivery and therapeutic capabilities of PtAuBNps. To the best of our knowledge, this is the first reported delivery of 5-FU with PtAuBNps, with the delivery of doxorubicin being reported previously [17]. 

## 2. Materials and Methods 

### 2.1. Materials

Hydrogen hexachloroplatinate(IV) hexahydrate (H_2_PtCl_6_.6H_2_O, Mw: 517.90 g/mol), gold (III) chloride trihydrate (HAuCL_4_.3H_2_O, Mw: 393.83 g/mol), polyvinylpyrrolidone (PVP, Mw 40,000), sodium borohydride (NaBH_4_, Mw: 37.83 g/mol), sodium triphosphate (Na_5_P_3_O_10_, Mw: 367.86 g mol^−1^), porcine mucin, sulphorhodamine B (SRB Dye, C_27_H_30_N_2_O_7_S_2_, Mw: 558.67 g/mol), 5-fluorouracil (5-FU, C_4_H_3_FN_2_O_2_, Mw: 130.1 g/mol), polysorbate 80 (Tween 80, Mw:1310 g/mol, C_64_H_124_O_26_), acridine orange hemi (zinc chloride) salt [3,6-Bis(dimethylamino) acridine hydrochloride zinc chloride double salt] (C_17_H_19_N_3_, Mw: 265.36 g/mol), chitosan (75% deacetylation) and dialysis tubing (MWCO ~ 12,000 Daltons) were purchased from Sigma-Aldrich (St. Louis, MO, USA). Ethidium bromide, glacial acetic acid, dimethyl sulphoxide (DMSO), 3-[(4,5-dimethylthiazol-2-yl)-2,5-diphenyl-2*H*-tetrazolium bromide] (MTT) and phosphate buffered saline tablets (PBS (140 mM NaCl, 10 mM phosphate buffer, and 3 mM KCl)) were sourced from Merck (Darmstadt, Germany). Eagle’s Minimum Essential Medium (EMEM) with l-glutamine (4.5 g/L), trypsin-versene-EDTA mixture and antibiotic mixture [penicillin (10,000 U/mL), streptomycin (10,000 μg/mL, and amphotericin B (25 μg/mL)] were purchased from Lonza BioWhittaker (Verviers, Belgium). Sterile foetal bovine serum (FBS) was purchased from Hyclone GE Healthcare (South Logan, UT, USA). Human embryonic kidney cells (HEK293), breast adenocarcinoma (MCF-7), human epithelial colorectal adenocarcinoma cells (Caco-2), and human hepatocellular carcinoma cells (HepG2) was obtained from the ATCC (Pty) Ltd., Manassas, VA, USA. All sterile tissue culture plasticware were obtained from Corning Inc. (Corning, NY, USA). All chemical reagents were of analytical grade and were used without further purification. Ultrapure (18 MOhm) water (Milli-Q50, Millipore, Molsheim, France) was used throughout. 

### 2.2. Preparation of Bimetallic PtAu Nanoparticles (PtAuBNps)

The PtAuBNps were prepared by concomitant chemical reduction of HAuCl_4_·3H_2_O and H_2_PtCl_6_·6H_2_O with NaBH_4_ in the presence of a PVP stabiliser [19]. Briefly, an aqueous solution of PVP (0.44 mM, 50 mL) was added to a HAuCl_4_·4H_2_O (25 mL, 0.44 mM) solution under gentle, constant stirring at 0 °C for 15 min. Thereafter, 25 mL of H_2_PtCl_6_·6H_2_O (0.4 mM) was added with stirring for 30 min at 0 °C. This was followed by a rapid injection of NaBH_4_ (6.67 mL, 16.5 mM, 0 °C) under vigorous stirring, until a dark brown colloidal suspension of PtAuBNps were formed. The final concentration of the synthesised BNps was 0.18 mg/mL. 

### 2.3. Preparation of Nanocomposites

The method used was modified from previous reports [19,20,21]. The anticancer drug 5-FU was encased within a Tween 80 stabilised CS based nanocomposite. Briefly, 0.75 mg/mL CS solution (in 2% glacial acetic acid) was added to 3.8 mM of 5-FU solution (in 18 Mohm water) in a 1:1 ratio (*v*/*v*) with constant mixing. Thereafter, Tween 80 (0.5% *v*/*v*) was added as a surfactant, and the pH adjusted to 4.8 with 0.1 M NaOH. The prepared drug–polymer solution was mixed with 1.4 mM TPP solution to obtain a CS: TPP ratio of (2:1 *v*/*v*). The nanoparticle suspension was gently stirred for 30 min to allow adsorption of 5-FU onto the CSNps (CTF). Finally, 0.18 mg/mL of colloidal PtAuBNps was added dropwise to the CTF nanoparticles at a ratio of (1:1 *v*/*v*) under gentle, constant stirring for 180 min, forming the nanocomposite, PtAu-CS-TPP-5FU/Tween 80 (PACTF). Finally, all drug loaded nanocomposites were purified by centrifugation at 15,000 rpm at 4 °C for 15 min (Beckman Ultracentrifuge, Brea, CA, USA) and the pellet re-dispersed in 18 Mohm water. 

### 2.4. Imaging, Nanoparticle Sizing and Zeta Potential Analysis 

The surface morphology, uniformity and size distribution of all nanoparticles (Nps) and nanocomposites were investigated using TEM (JEOL JEM 1010, Tokyo, Japan, functioning at 100 kV). Aqueous solutions of Nps/nanocomposites were deposited onto separate formvar coated 40-mesh copper grids (Ted Pella Inc., Redding, CA, USA), and air dried. Images were recorded using the iTEM Soft Imaging Systems (SIS) Megaview III (JEOL JEM 1010, Tokyo, Japan) fitted with a side-mounted 3-megapixel digital camera.

The particle size distribution, concentration and colloidal stability were measured by nanoparticle tracking analysis (NTA, Nanosight NS500; Malvern Instruments, Malvern, Worcestershire, UK). All PtAuBNp formulations were diluted 1:1000 in 18 Mohm water and run in triplicates. Individual particles undergoing Brownian motion were captured and visualised through light scattering upon laser illumination. The NTA software measures the theoretical hydrodynamic diameter of particles by application of the Stokes–Einstein equation and the zeta (ζ) potential by Laser-Doppler microelectrophoresis through Smoluchowski modelling. All measurements were performed at 25 °C and 24 V.

### 2.5. UV-Vis Spectrophotometry 

The confirmation of the PtAu core–shell formation, successful chitosan polymerisation and 5-FU entrapment was based on the optical changes of specific samples and verified against that in literature. Briefly, solutions of approximately 10 µL were analysed over a wavelength range of 200–800 nm using a UV-vis spectrophotometer (JASCO V-730, JASCO Corporation, Hachioji, Japan).

### 2.6. Fourier Transform Infra-Red (FTIR) Spectroscopy 

To identify surface-bound functional groups, and the chemical interactions between the carrier and the drug, FTIR analysis were conducted in a Perkin Elmer spectrum 100 FTIR spectrometer equipped with a diamond universal ATR sampling accessory. The spectra were acquired at a programmed range of 400–4000 cm^−1^ at a 1 cm^−1^ resolution. The spectra requisition was carried out using the Spectrum 10 analysis software (Perkin Elmer, Waltham, MA, USA).

### 2.7. Drug Binding Studies 

The drug entrapment efficiency (EE) and loading content (LC) were estimated by the amount of drug liberated after centrifugation. Briefly, drug laden nanocomposites were centrifuged at 21,000 rpm for 15 min at 10 °C (Beckman Ultracentrifuge, Brea, CA, USA), to separate the bound and unbound drug. The analysis of unbound or free 5-FU in the supernatant was determined by UV-vis spectroscopy at a wavelength 481 nm. Measurements were conducted in triplicate. The theoretical drug content (TDC), encapsulation efficiency (EE), actual drug content and loading capacity (LC) were calculated using Equations (1)–(4) [17].

(1) $$\mathrm{TDC}=\frac{\mathrm{Weight}\;\mathrm{of}\;5-\mathrm{FU}}{\mathrm{Weight}\;\mathrm{of}\;\mathrm{nanocomposite}}$$

(2) $$\mathrm{EE}\;\left(\%\right)=\frac{\mathrm{Total}\;5-\mathrm{FU}\;\mathrm{added}-\mathrm{Free}\;5-\mathrm{FU}}{\mathrm{Total}\;5-\mathrm{FU}\;\mathrm{added}}\times 100$$

(3) ADC (μg)=TDC×EE (%)

(4) $$\mathrm{LC}\;\left(\%\right)=\frac{\mathrm{Total}\;5-\mathrm{FU}\;\mathrm{added}-\mathrm{Free}\;5-\mathrm{FU}}{\mathrm{weight}\;\mathrm{of}\;\mathrm{nanocomposite}}\times 100$$

### 2.8. In Vitro Mucoadhesive Response

The bioadhesive propensity of the nanocomposites was studied in vitro as a means of the rheological synergism that occurs at the functional group level between nanoparticles/nanocomposites and a porcine mucin model. Approximately, 1 mL of porcine mucin (PM, 400 µg/mL) suspension in simulated intestinal fluid (25% *v*/*v*, pH 6.8) was mixed with 1 mL of the respective nanocomposite suspensions (20 µg/mL), and gently shaken (50 rpm) at 37 °C for 6 h. Thereafter, free PM was separated by centrifugation at 21,000 rpm for 30 min at 10 °C (Eppendorf 5424R, Hamburg, Germay). The degree of interaction between the nanocomposites and mucin was determined by measuring the absorbance of the remaining free PM in the supernatant in a spectrophotometer set at wavelength of 251 nm, with intestinal fluid as the blank. The percentage mucoadhesion was calculated using Equation (5).

(5) $$\mathrm{Mucoadhesion}\;\left(\%\right)=\frac{\mathrm{Total}\;\mathrm{Mucin}\;\mathrm{before}-\mathrm{Free}\;\mathrm{Mucin}\;\mathrm{after}\;}{\mathrm{Total}\;\mathrm{Mucin}\;\mathrm{before}}\times 100$$

### 2.9. Pharmacokinetic Studies 

The ability of the prepared nanocomposites to release the loaded drug in response to specific biological environments was investigated. Approximately, 5 mL of drug loaded nanocomposites (50 µg/mL) were placed in dialysis bags (MWCO 2000 Da), hermetically sealed and immersed separately in 10 mL of PBS (pH 7.4, 6.5, 5.0 and 4.5), with gentle stirring at 37 °C. Periodically, 10 µL aliquots were withdrawn and analysed. The amount of 5-FU released was determined using UV-vis spectroscopy at 266 nm. The cumulative drug release (%) was calculated relative to the total absorbance of 5-FU loaded onto the nanocomposites using Equation (6).

(6) $$\mathrm{Cumulative}\;\left(\%\right)=\frac{\mathrm{Abs}\;\mathrm{of}\;\mathrm{free}\;5-\mathrm{FU}}{\mathrm{Abs}\;\mathrm{of}\;\mathrm{total}\;5-\mathrm{FU}\;\mathrm{loaded}}\times 100$$

The drug release data were modelled using the zero-order, first-order, Higuchi’s square root of time equation and the Korsmeyer–Peppas power law kinetic equations [22,23,24] (Table 1). 

### 2.10. In Vitro Cytotoxicity Assessment 

All cells were cultured in EMEM supplemented with 10% FBS and 1% antibiotics. The cells were maintained in 25 cm^2^ culture flasks under standard culture conditions (37 °C, 5% CO_2_ and 95% relative humidity), and were sub-cultured routinely. All biological assays were conducted under aseptic conditions in an Airvolution Class II biosafety laminar flow hood (United Scientific, Western Cape, South Africa).

The antitumour activities of PtAuBNps and their nanocomposites were evaluated in vitro using the MTT and SRB assays in three human cancer cell lines (Caco-2, HepG2 and MCF-7) and a non-cancer cell line (HEK293). Exponentially growing cells were trypsinised, seeded in a 96-well plate at a cell density of 2.5 × 10^3^ cells/well and incubated overnight at 37 °C. Thereafter, spent medium was replenished with 100 µL fresh growth medium, to which the respective compounds were added at various concentrations (5, 15, 35 and 50 µg/mL), and incubated for 48 h at 37 °C. Wells containing cells only served as the positive control. All assays were done in triplicate. The MTT and SRB assays were conducted as described below, after the 48-h incubation period. The cell viability (%) in each of the assays was calculated using Equation (7).

(7) $$\mathrm{Cell}\;\mathrm{viability}\;(\%)=\frac{\mathrm{Abs}\;\mathrm{of}\;\mathrm{treated}\;\mathrm{cells}}{\mathrm{Abs}\;\mathrm{of}\;\mathrm{untreated}\;\mathrm{cells}}\times 100$$

For the MTT assay, the culture medium was aspirated and replenished with 100 µL of EMEM containing 10% MTT reagent (5 mg/mL in PBS), and incubated for 4 h at 37 °C. Thereafter, the MTT-medium solution was carefully removed, and 100 µL DMSO added to each well to ensure cell permeation and solubilisation of the formazan sediment. Absorbance was read using a Mindray MR-96A microplate reader (Vacutec, Hamburg, Germany) at 570 nm, with DMSO as a blank. 

For the SRB assay, the cell monolayers were fixed by gently layering 25 µL cold TCA (50% *w*/*v*) onto the growth medium. The cells were incubated for 1 h at 4 °C, washed (3×) with distilled water and air dried. The TCA-fixed cells were then stained with 50 µL of SRB (0.4% *w*/*v* in 1% glacial acetic acid) dye for 30 min at 37 °C, washed (3×) with 1% acetic acid to remove the non-ligated dye, and the plates air dried. Finally, the protein-bound dye was extracted with 100 µL of Tris buffer (10 mM, pH 10.5), and absorbances measured at 565 nm using Tris base as the blank. 

### 2.11. Apoptosis Assay 

The acridine orange/ethidium bromide (AO/EB) dual staining method is a convenient, rapid and economical method for the quantitative and qualitative analysis of possible apoptosis induction by the drug laden PtAuBNps. Cells were seeded at a cell density of 1.5 × 10^5^ in a 24-well plate and incubated at 37 °C in 5% CO_2_ for 24 h, to allow the cells to attach. Thereafter, the culture medium was aspirated, replenished with 0.5 mL of complete medium, and cells were treated with nanocomposites at predetermined IC_50_ values (average of the two assays). A nanoparticle/nanocomposite free positive control was included. After a 24 h incubation at 37 °C, the spent medium was removed, and cells washed (2×) with 100 µL of cold PBS. Cells were stained with 12 µL of the dye solution (1:1 *v*/*v* AO: EB, 100 mg/mL in PBS) for 5 min. Cells were viewed under an Olympus fluorescent microscope (200× magnification), fitted with a CC12 fluorescent camera (Olympus Co., Tokyo, Japan). The number of viable cells and apoptotic bodies were tallied using the Soft Imaging System (SIS) software (Olympus Co., Tokyo, Japan). The apoptotic indices were calculated according to Equation (8).

(8) $$\mathrm{Apoptotic}\;\mathrm{Index}=\frac{\mathrm{Number}\;\mathrm{of}\;\mathrm{Apoptotic}\;\mathrm{cells}}{\mathrm{Total}\;\mathrm{number}\;\mathrm{of}\;\mathrm{cells}\;\mathrm{counted}}$$

### 2.12. Statistical Analysis

The results in triplicate are reported as mean ± SD (standard deviation). All statistical analyses were performed using GraphPad Prism version 5.01 (GraphPad Software Inc., La Jolla, CA, USA). The significance of results and differences between the control and test were determined using a one-way analysis of variance (ANOVA). The Dunnett’s post hoc test was used for the growth inhibition assays. Statistical significance between groups was considered significant at ** *p* < 0.01 and * *p* < 0.05. Dissolution kinetics parameters were evaluated using Microsoft Excel 2016^TM^ and DD Solver software. The parameters are indicated in Table 1. The best-fit dissolution profile was identified at r^2^ values ≥ 0.99.

## 3. Results

### 3.1. Nanoparticle Morphology, Sizing and Zeta Potential 

**Figure 2:**
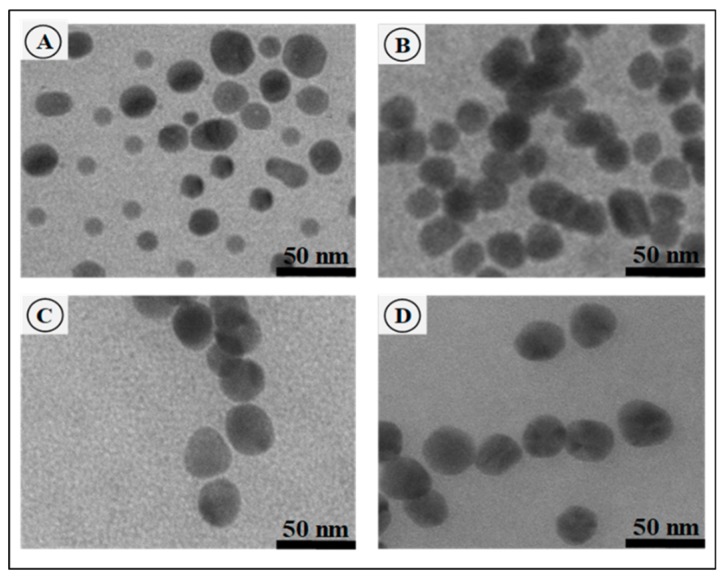
TEM micrographs of: (**A**) PtAuBNps; (**B**) PtAuCSBNps; (**C**) CTF; and (**D**) PACTF. Bar = 50 nm. CTF, CS-TPP-5-FU/Tween 80; PACTF, PtAu-CS-TPP-5-FU/Tween 80.

TEM reflected the ultrastructural morphology, distribution and uniformity of all nano-formulations (Figure 2A–D). The PtAuBNps (Figure 2A) displayed a near spherical morphology and were well dispersed due to passivation with PVP, as observed in the literature [19,20,21]. All CS based nano-formulations (Figure 2B–D) were predominantly monodispersed, uniform spherical nanostructures with smooth surfaces. An increase in size for the drug bearing nanocomposites CTF (Figure 2C), and PACTF (Figure 2D) was evident, with the latter appearing denser and more compact. 

NTA analysis (Table 2) strongly correlated with the TEM results, and revealed PtAuBNps to have a hydrodynamic size of 69.9 ± 3.2 nm and a zeta (ζ) potential of −21.5 mV. Conjugation of CS to PtAuBNps increased the average hydrodynamic size to 88.4 ± 10.8 nm, with a shift from a negative to a highly positive ζ potential value (58.2 ± 1.1 mV). 

### 3.2. UV-Vis and FTIR Spectroscopy 

**Figure 3:**
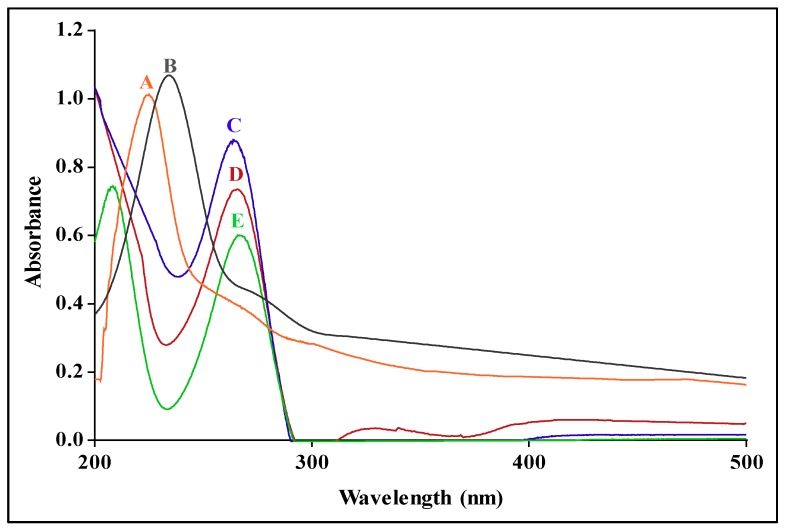
UV-vis spectra of: (**A**) PtAuBNps; (**B**) PtAuCSBNps; (**C**) PACTF; (**D**) CTF; and (**E**) 5-FU. 5-FU, 5-Fluorouracil; CTF, CS-TPP-5-FU/Tween 80; PACTF, PtAu-CS-TPP-5-FU/Tween 80.

The SPR of the PtAuBNPs (Figure 3A) presented as a single narrow peak centred at 224 nm, correlating with the literature and confirming the successful synthesis of core–shell nanostructures [25]. Chitosan functionalised PtAuBNps (Figure 3B) exhibited a single broad red shift with an SPR resonant extinction peak at 235 nm, indicating a change in the local refractive index due to successful polymer conjugation and an increase in particle size. Successful encapsulation and loading of 5-FU were confirmed in the nanocomposites PACTF and CTF (Figure 3C,D). The characteristic absorbance peak of 5-FU at 266 nm (Figure 3E) displayed a blue shift to 261 nm and 263 nm for PACTF and CTF, respectively, correlating to the literature [16]. 

**Figure 4:**
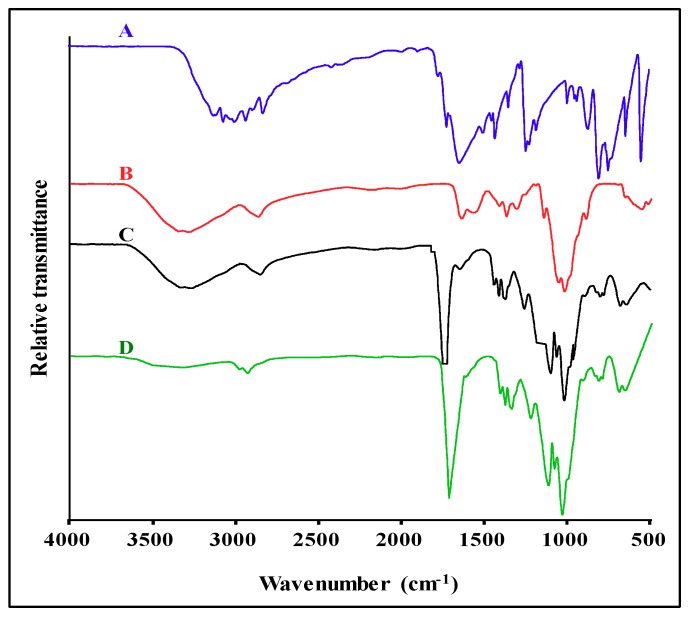
FTIR Spectra of: (**A**) 5-FU; (**B**) CS; (**C**) PACTF; and (**D**) CTF. CTF, CS-TPP-5-FU/Tween 80; PACTF, PtAu-CS-TPP-5-FU/Tween 80.

The FTIR spectrum of 5-FU (Figure 4A) displayed the characteristic vibrational bands within 3000–2825 cm^−1^, attributed to C–H stretching. Absorption bands at ~1726 cm^−1^ and 1669 cm^−1^ are ascribed to C=O and N–H vibrations, respectively. Bands at ~1428–1504 cm^−1^ are due to C=C and C=N stretching. The band at ~1242 cm^−1^ is due to C-N vibrations [2,26]. The CS spectra (Figure 4B) exhibited characteristic O-H and N-H stretching bands at ~3352 cm^−1^, stretching vibrations of the C-H bond at ~2935 cm^−1^, C=O stretching of the amide I band at ~1647 cm^−1^, vibrations of the N–H group of the amide II band at ~1573 cm^−1^, anti-symmetric stretching of the (C–O–C) bridge at ~1150 cm^−1^, and a NH_2_ peak at ~895 cm^−1^, all corresponding to that reported in the literature [27,28]. Drug loaded nanocomposites (Figure 4C,D) displayed most of the signature stretching and deformation vibrations of CS and 5-FU with minor shifts. However, the 5-FU footprints were slightly masked and diminished by the nanoparticle spectra, suggesting successful 5-FU encapsulation. 

### 3.3. Drug Binding Studies 

The encapsulation efficiency (EE) and loading content (LC) of 5-FU was 90.17% and 22.56% in PACTF, with a lower EE of 87.24% and a higher LC of 23.24% in CTF (Table 3). 

### 3.4. In Vitro Mucoadhesive Response

CS functionalised PtAuBNps exhibited the highest porcine mucin (PM) binding efficiency (86.24 ± 3.82%) (Table 4), while the free drug and PtAuBNps, showed lower mucoadhesive ability, possibly due to charge repulsions. Nanocomplexes, PACTF (68.74 ± 2.87%) and CTF (67.05 ± 4.21%), demonstrated a slightly lower degree of mucoadhesion than the PtAuCSBNps, possibly due to the utilisation of the free surface amino groups through TPP cross-linking, resulting in weaker binding interactions. 

### 3.5. In Vitro Pharmacokinetics Studies 

**Figure 5:**
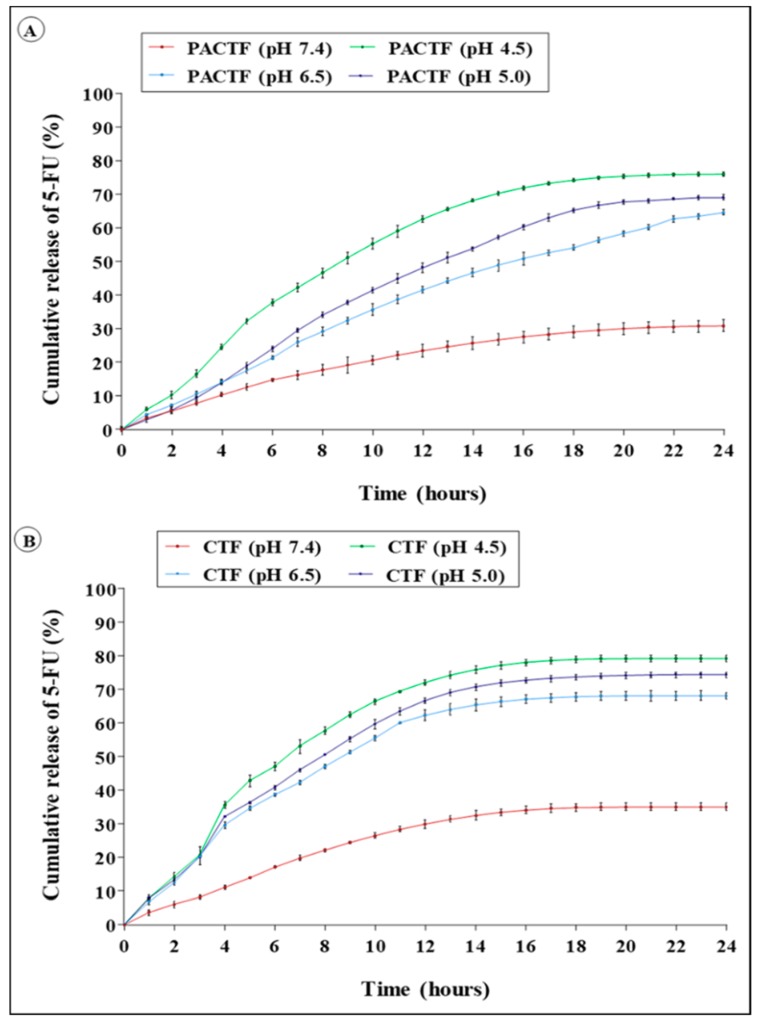
In vitro cumulative drug release profile of 5-fluorouracil encapsulated nanocomposites at pH 4.5, 5.0, 6.5 and 7.4: (**A**) PACTF; and (**B**) CTF.

The pharmacokinetic profiles PACTF and CTF (Figure 5 and Tables S1 and S2), show that the release of 5-FU was slow at neutral conditions for CTF (35.11% ± 1.17 in 24 h) and PACTF (30.80 ± 1.75 in 24 h), but accelerated at lower pH. A rapid release was observed at pH 4.5 with up to 70% of 5-FU eluted over the 24 h period. Approximately, 54.0 ± 1.8%, 65.2 ± 0.66% and 74.1 ± 0.88% were eluted from PACTF at pH 6.5, 5.0 and 4.5, respectively. At neutral milieu, the release from both nanocomposites closely followed the zero-order kinetic model with limited dissolution of 5-FU by non-Fickian diffusion, while release under acidic conditions followed the zero-order model (r^2^ = 0.977). However, the liberation of 5-FU from CTF and PACTF occurred through anomalous diffusion. 

### 3.6. In Vitro Cytotoxicity 

**Figure 6:**
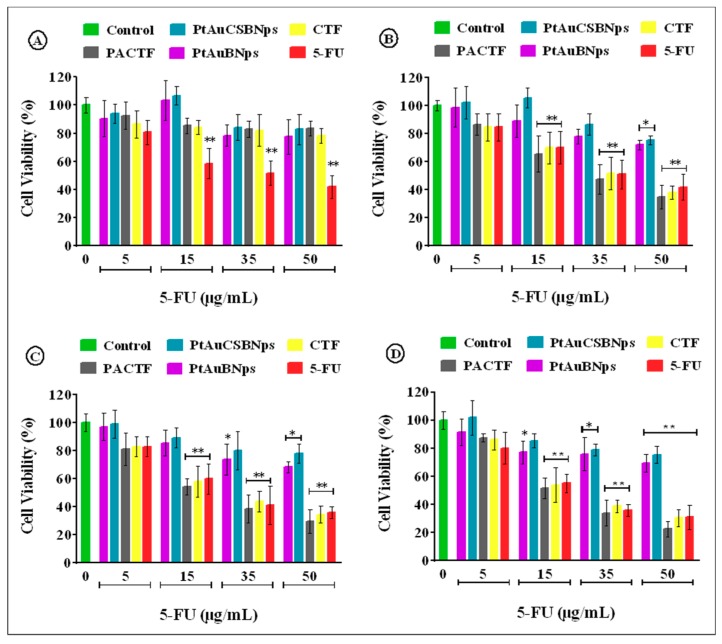
MTT cytotoxicity assay of bimetallic nanoparticles and drug bearing nanocomposites in: (**A**) HEK293; (**B**) MCF-7; (**C**) HepG2; and (**D**) Caco-2 cell lines. Data are presented as mean ± SD (*n* = 3). * *p* < 0.05, ** *p* < 0.01 were considered statistically significant.

**Figure 7:**
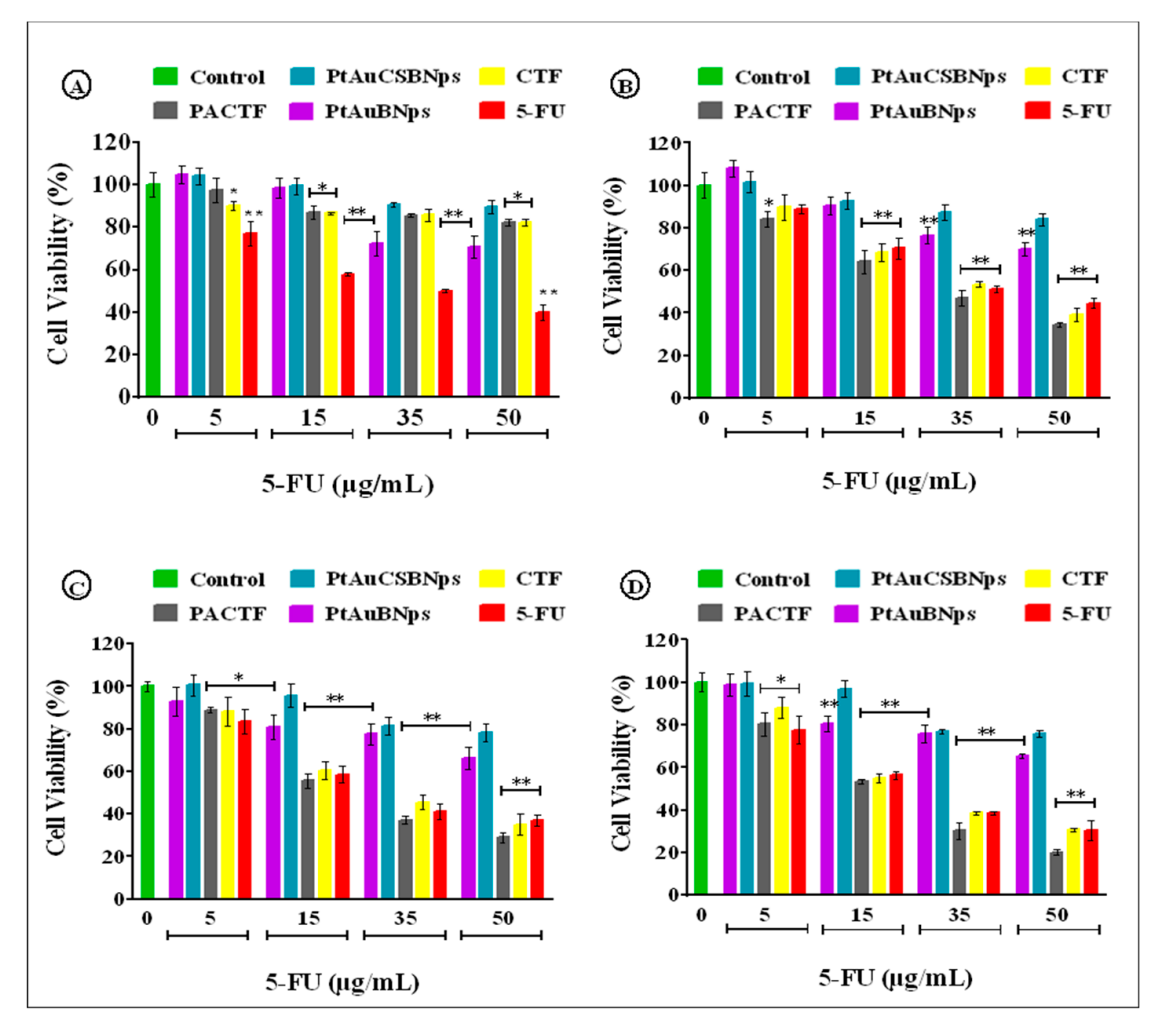
SRB cytotoxicity assay of bimetallic nanoparticles and drug bearing nanocomposites in (**A**) HEK293; (**B**) MCF-7; (**C**) HepG2; and (**D**) Caco-2 cell lines. Data are presented as mean ± SD (*n* = 3). * *p* < 0.05, ** *p* < 0.01 were considered statistically significant.

The cytotoxicity profiles and IC_50_ values of PtAuBNPs, PtAuCSBNPs, CTF and PACTF (Figure 6 and Figure 7 and Table 5), showed that the PtAuBNps exerted low cytotoxicity at the highest tested concentration, with up to 75% maximum cell viability in all cell lines in both assays. 

### 3.7. Apoptosis Induction Studies 

**Figure 8:**
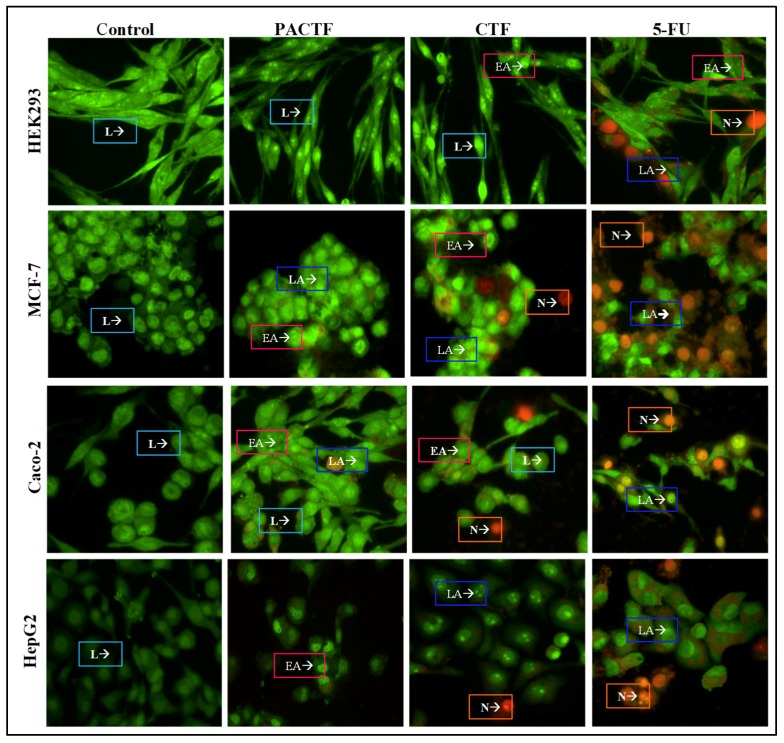
Fluorescence images of dual acridine orange/ethidium bromide stained cells showing induced morphological changes in HEK293, MCF-7, Caco-2 and HepG2 cell lines at 20× magnification. L, Live cells; EA, Early apoptotic cells; LA, Late apoptotic cells N, Necrotic cells.

The fluorescent images of the control and treated cells are depicted in Figure 8, and the apoptotic index (AI) in Table 6. All control cells emitted green fluorescence indicative of healthy cells with an intact cell membrane. Conversely, all cells treated with 5-FU, PACTF and CTF at their IC_50_ values, formed apoptotic bodies of varying degrees in all cell lines. 5-FU induced high degrees of cell death in all cell lines through both apoptotic and necrotic pathways. The encapsulation of 5-FU brought about biocompatibility and controlled cell death as displayed by the very low apoptotic indices of the nanocomposites in the HEK293 (<0.052) cells, and the considerably higher indices in the three cancer cell lines (>0.360). The MCF-7 cell line showed cells mainly in early apoptosis, while the Caco-2 and HepG2 cells were more sensitive with higher apoptotic indices and characteristic apoptotic features (membrane blebs, chromatin condensation and fragmentation). 

## 4. Discussion

The morphological features and colouration of the CSNps under TEM were similar to previously reported findings [17,29,30]. Characteristically, metallic nanoparticles with high atomic numbers possess excellent light scattering power and appear dark in colouration [31]. Nanocomposite PACTF appeared to have areas of dark pigmentation, suggesting the presence of small-sized PtAuBNps within dense chitosan cross-linked nanospheres. 

Zeta (ζ) potential is the magnitude of the electrostatic potential generated on the edge of the slipping plane between the particle and the dispersant medium. In general, particles displaying a ζ potential value >30 mV or <−30 mV, will have a strong degree of electrostatic repulsion between adjacent similarly charged particles, leading to better colloidal dispersion [32]. However, a ζ potential <15 mV or >−15 mV, will have attractive forces that exceed repulsive forces, causing particles to aggregate. Conjugation of CS to PtAuBNps increased the average hydrodynamic size to 88.4 ± 10.8 nm, with a shift from a negative to a highly positive ζ potential value (58.2 ± 1.1 mV). This finding was in keeping with similar analysis conducted on CS functionalised AuNps [17,33,34,35]. The change in surface potential for PtAuCSBNps suggest strong electrostatic interactions between the protonated CS and the negatively charged surface of the PtAuBNps. Such high surface potential imparts a high degree of colloidal stability, to facilitate efficient cellular uptake by the negatively charged cellular membrane. The loading of 5-FU brought about an increase in size to 118.8 ± 8.6 nm for CTF and 108.6 ± 8.2 nm for PACTF. Furthermore, CTF (28.3 ± 2.6 mV) and PACTF (30.5 ± 0.6 mV) had lower ζ potentials than PtAuCSBNps, which can be ascribed to the consumption of free amine groups through TPP cross-linking and drug encapsulation, corroborating FTIR findings. The smaller hydrodynamic size of PACTF is possibly due to the addition of PtAuBNps, which condensed the CS framework. Previous studies on these bimetallic nanoparticles encapsulating the drug, doxorubicin, showed similar increases in size and zeta potential [17]. However, the sizes of these 5-FU nanocomplexes were slightly larger with marginally lower zeta potentials. Studies with functionalised gold nanoparticles showed a similar trend forming larger nanocomplexes but with high zeta potentials [35]. No studies using functionalised platinum nanoparticles for 5-FU delivery were found for comparison. Overall, these investigated nanocomplexes displayed physiochemical properties deemed to be critical for better tissue penetration, long-term storage and enhanced therapeutic effects. 

Spherical AuNps dispersed in water display a strong SPR absorbance band in the visible region (520 nm), and PtNps in the near-infrared region (220 nm), which is attributed to in-plane dipole resonance [36]. The formation of Au core–Pt shell nanostructures is accompanied by a blue shift and quenching of AuNps’ SPR, as Pt shell atoms epitaxially nucleate and grow on the surface of the Au core, until there is complete disappearance of the AuNps SPR [37]. The red shift following polymer conjugation has been previously reported [35,38,39]. FTIR is a well-established technique to identify and confirm the functional groups present by the magnitude, relative intensity and shape of the absorption bands that arise through stretching and deformation vibration. FTIR spectroscopy confirmed the chemical structure and functional groups present in CS, PtAuCSBNp, PACTF, CTF and 5-FU and corroborated that of UV-vis spectroscopy.

The results of drug encapsulation suggest that there is a strong correlation between ζ potential analysis and EE, with a higher ζ potential in PACTF relating to a higher EE, while the compactness possibly resulted in the lower LC. These findings were consistent with similar 5-FU binding studies conducted with CS/Au nanocomposites [26]. Our previous studies, using doxorubicin containing PtAuCSBNps with slightly lower ζ potentials, produced lower drug encapsulation averaging around 70% [17], further confirming the correlation between ζ potential and EE. Furthermore, EE of 74% and 79% of 5-FU was reported for CS functionalised and folate-CS functionalised gold nanoparticles which displayed higher ζ potentials (>50 mV), which was still lower than that recorded for PACTF in this study. Although this encapsulation efficiency for the PtAuCSBNps was good, there is a need to improve its biostability for enhanced therapeutic effect especially for in vivo applications. The development of a bioadhesive drug delivery system has the potential to increase the residence time at the application site, increase drug permeation and bioavailability. Our findings support the notion that the positively charged amine groups of CS are mainly responsible for the carrier’s bioadhesive propensity [40]. This phenomenon occurs specifically at the molecular level, through electrostatic interactions between the positively charged amino groups of CS and the negatively charged sulphonic acid resides in the mucin, bringing about rheological synergism [41,42,43]. Hence, this system has the potential to pass through the mucosal layer, enhancing its attractiveness as a drug delivery system.

Tumour tissue presents a mildly acidic microenvironment (pH 6.5–5.0) due to vascular irregularities, hypoxia and high glycolytic metabolism, leading to the production and accumulation of acidic metabolites, with lower acidity occurring in intracellular organelles, viz., endosomes and lysosomes (pH 5.5–4.0) [44,45]. Recently, pH-responsive release systems have emerged as attractive strategies to selectively target tumour acidity, enhance the therapeutic index and reduce side effects by providing spatiotemporal control over drug release in the body [46,47]. CTF displayed a characteristic biphasic release pattern at acidic environments, comprised of an initial release surge over the first 10 h, followed by a slow gradual release of 5-FU in a plateau phase for the subsequent 14 h. This release behaviour has previously been reported for polymeric nanoparticles [48,49]. The 24 h release percentages of CTF were approximately 68.2 ± 1.0%, 74.4 ± 0.9% and 79.2 ± 0.97% at pH 6.5, 5.0 and 4.5, respectively. The initial burst displayed in the dissolution profile of CTF can be attributed to release of the surface-associated drug, while the long plateau phase is probably due to release of the encapsulated drug within the dense polymeric matrix [50]. PACTF showed a limited burst release at acidic conditions (pH 6.5, 5.0 and 4.5), providing a gradual release for 18 h and a slow 6 h release phase, demonstrating better encapsulation of 5-FU within the core of the PACTF nanostructure. The pH-sensitive behaviour of PACTF and CTF can be attributed to conformational changes that take place in response to variations in physiological pH. At neutral conditions, CS remains stable/deprotonated, while at acidic conditions the protonation of the pendant amine groups causes the carriers to undergo gel-sol transitions, swelling and release of the encapsulated drug into the bathing liquid [51].

Kinetic modelling of the release data further characterised the dissolution of 5-FU from the nanocomplexes. The dialysis method is affected mainly by water imbibition, drug diffusion and polymer dissociation [52]. The exponent n of the Korsmeyer–Peppas model provides valuable insight into the release mechanism as Fickian diffusion (0.45 ≤ *n*), non-Fickian (anomalous) diffusion (0.45 < *n* < 0.89), case II transport (*n* = 0.89) or super case II transport (*n* > 0.89) [24]. Findings suggest that 5-FU encapsulated in CS based nano-formulations was retained, and released slowly over a prolonged period through a combination of diffusion and polymer erosion. 

The MTT assay provided an estimation of cell viability centred on the principle that only metabolically active cells can convert MTT into a purple insoluble formazan product, while the SRB assay estimates cell number by staining TCA fixed cellular proteins with the pink aminoxanthine SRB dye [53]. Similar trends in cytotoxicity for both assays suggest a strong correlation between the two colorimetric assays. The PtAuCSBNps displayed no relevant in vitro cytotoxicity and even stimulated the growth of the HEK293 cells, suggesting good biocompatibility, with the CS moieties possibly serving as a nutrient source, similar to previous studies incorporating CS with nanoparticles conducted by our group [17,54]. Exposure to free 5-FU elicited a dose dependent decrement of cell survival to less than 45% in all cell lines, similar to previous reports [26,55,56]. Nanocomposites (PACTF and CTF) were well tolerated in the HEK293 cell line with more than 75% maximum cell viability, but inflicted significantly greater damage to all cancer cell lines compared to the 5-FU at similar concentrations. The most profound antiproliferative effects were seen in the Caco-2 cells with up to 30% cell viability at the highest tested dosage. Similar results were obtained in our previous studies using these bimetallic nanoparticles to deliver doxorubicin [17], where the drug nanocomplexes exhibited high activity in the MCF-7 and Caco-2 cells at lower concentrations, as evidenced in the low IC_50_ values obtained. The doxorubicin nanocomplexes however were slightly larger than the 5-FU nanocomplexes in this study but were equally stable. Hence, a similar trend in the cytotoxicity profiles was obtained, suggesting that the PtAuBNps played a significant role in the cellular uptake and overall activity of the drugs in vitro. The IC_50_ values of PACTF and CTF for the Caco-2 cells were approximately 18.98 µg/mL and 21.99 µg/mL in MTT assay, and 19.25 µg/mL and 22.58 µg/mL in the SRB assay. In the HepG2 cell line, PACTF and CTF displayed slightly higher IC_50_ values, of approximately 22.85 µg/mL, and 25.21 µg/mL in the MTT assay, and 23.10 µg/mL and 26.24 µg/mL in the SRB assay. PACTF and CTF were least effective in the MCF-7 cell line with high IC_50_ values of 29.73 µg/mL and 33.57 µg/mL in the MTT assay, and 30.12 µg/mL and 34.99 µg/mL in the SRB assay. The toxicity profiles obtained clearly support enhanced cytotoxicity after 5-FU loading. PACTF had the best anticancer activity in vitro in all cancer cell models, with excellent tolerance in the non-cancer HEK293 cell line. This can be attributed to the good stability (zeta potential of 30.5 mV) of PACTF favouring cellular uptake, its high 5-FU encapsulation (~90%), and importantly its 5-FU release profile (Figure 5), which showed greater release of the drug (65–74%) at lower pHs (6.5, 5.0, 4.5), compared to the release (~20%) at physiological pH 7.4. Hence, 5-FU had a greater effect on the cancer cells than the non–cancer HEK293 cells. Hence, it can be inferred that the addition of PtAuBNps acted synergistically with 5-FU to enhance cytotoxicity, and were in consonance with the physiochemical characterisations and drug release profiles. 

Programmed cell death or apoptosis is a regulatory mechanism for the removal of physiologically defective, unwanted, damaged or dysfunctional cells [57,58]. Cells undergoing apoptosis display distinct morphological features that include cytoplasmic shrinkage, chromatin condensation, loss of membrane phospholipid asymmetry, DNA fragmentation and membrane blebbing [57,58,59,60]. Acridine orange permeates all cells resulting in the emittance of green fluorescence, whereas ethidium bromide is only taken up by non-viable cells that have lost their cytoplasmic membrane integrity causing the nucleus to fluoresce red. The nucleus of viable cells emits a green fluorescence, early apoptotic cells with condensed or fragmented chromatin bright green, late apoptotic with condensed and fragmented chromatin yellow/orange and necrotic cells with no condensed chromatin orange/red [61]. Overall, the apoptosis studies corroborated well with growth inhibition (MTT and SRB assay), and drug release studies, with the drug nanocomplexes exhibiting a pH-sensitive release, especially PACTF which based on the kinetic modelling showed a steady release of the drug with no sudden burst release, and a zero-order release profile for enhanced therapeutic effects. This zero-order release profile also assists in determining the bioavailability of the drug, thereby enhancing the cellular uptake and treatment strategy [35]. The higher release at lower pHs may be significant in producing a system with greater activity in tumour micro-environments than under normal physiological conditions. This cancer cell targeting based on pH sensitivity may be further assisted by active receptor targeting the cancer cells, such as reported for folate targeting of 5-FU using polymerized gold nanoparticles [35].

The two metals, gold and platinum were combined to determine if the favourable properties of both metals would act synergistically producing a potentially efficient nanoparticle for drug delivery. This initial study was solely conducted in vitro and we are currently looking at further research using in vivo systems. On the positive side, this study did show some pH responsive behaviour of the nanocomplexes suggesting that they may target the cancer cells due to their low pH micro-environment. However, further studies and optimizations are required to produce a more definitive conclusion. This bimetalic system was shown to be promising, and being the first one reported for the delivery of 5-FU, it can be used to lay the foundation for future such research which may exceed the current strategy. Despite the vast array of existing systems, there is always a need for a more robust and stable delivery system. Future persepectives, can include the varying of the core-shell composition, use of alternate polymers such as polyethylene-glycol and poly(lactide-*co*-glycolide), and the introduction of targeting ligands for cancer cell specific delivery.

## 5. Conclusions

A novel, multifunctional, practical, customisable, stable, nanosized and pH-responsive PtAuCS bimetallic delivery system, exhibiting significantly higher anticancer potency than free 5-FU alone was established in this preclinical study. The embedding of PtAuBNps with CSNps acted synergistically with 5-FU to enhance its in vitro cytotoxicity, offering the prospects of reducing drug concentration and the frequency of dosage. Furthermore, this study strongly supports the notion that pH-triggered drug release brings about site-specific toxicity and enhances intracellular bioaccumulation of drugs, such as 5-FU that require metabolic activation to exert its cytotoxic effects. Overall, the PtAuCS bimetallic platform was shown to display superior optical properties, physiochemical features, pharmacokinetics, drug encapsulation, mucoadhesion and biocompatibility compared to the polymeric CSNps and the free 5-FU. These favourable physiochemical and biological attributes augur well for future in vivo and clinical applications, especially in the area of cancer therapy. Further studies would also involve the conjugation of a targeting moiety for cell specific delivery.

## Figures and Tables

**Table 1:** Time-dependent pharmacokinetic modelling of dissolution data to ascertain drug release mechanisms at acidic and physiological pH conditions.

Kinetic Model	Equation
Zero-order	*R*_t_ = *R*_0_ + *K*_0_*t*
First-order	Ln *R*_t_ = ln*R*_0_+ *K*_1_*t*
Higuchi	*R*_t_ = *K*_H_*t*^1/2^
Korsmeyer–Peppas	*R*_t/_*R*_∞_ = *K*_k_*t^n^*

*K*_0_, *K*_1_, *K*_H_, and *K*_k_ are release rate constants; *n* is the release exponent (indicative of drug release mechanism); *R*_0_ is the initial amount of 5-FU in the nanocomposite; *R*_∞_ is the total amount of drug dissolved when the dosage form is exhausted; and *R*_t_ is the amount of 5-FU released at time *t*.

**Table 2:** Size distribution and zeta potential of BNps and nanocomposites. Data are represented as mean ± SD (*n* = 3).

Sample	Particle Size (nm)	ζ Potential (mV)
PtAuBNps	69.9 ± 3.2	−21.5 ± 1.4
PtAuCSBNps	88.4 ± 10.8	58.2 ± 1.1
PACTF	108.6 ± 8.2	30.5 ± 0.6
CTF	118.8 ± 8.6	28.3 ± 2.6

CTF, CS-TPP-5-FU/Tween 80; PACTF, PtAu-CS-TPP-5-FU/Tween 80.

**Table 3:** Drug loading efficiency, theoretical drug content, actual drug content and drug loading content of nanocomposites.

Sample	TDC (µg)	EE (%)	ADC (µg)	LC (%)
CTF	139.21	87.24	121.45	23.24
PACTF	96.34	90.17	88.80	22.56

ADC, Actual drug content; CTF, CS-TPP-5-FU/Tween 80; EE, Encapsulation efficiency; LC, Drug loading content; PACTF, PtAu-CS-TPP-5-FU/ Tween 80; TDC, Theoretical drug content.

**Table 4:** Binding efficiencies of nanoparticles/nanocomposites to porcine mucin.

Compound	Mucoadhesion (%)
PtAuBNps	8.72 ± 1.67
PtAuCSBNps	86.24 ± 3.82
PACTF	68.74 ± 2.87
CTF	60.05 ± 4.21
5-FU	21.51 ± 3.28

Data are represented as mean ± SD (*n* = 3). 5-FU, 5-Fluorouracil, CTF, CS-TPP-5-FU/Tween 80; PACT, PtAu-CS-TPP-5-FU/Tween 80.

**Table 5:** IC_50_ values of free 5U and 5-FU loaded nanocomposites in HEK293, HepG2, Caco-2 and MCF-7 cell lines for the MTT and SRB assays.

Cell Lines	IC_50_ (µg/mL)—MTT Assay			IC_50_ (µg/mL)—SRB Assay		
	PACTF	CTF	5-FU	PACTF	CTF	5-FU
HEK293	-	-	31.79	-	-	30.84
MCF-7	29.73	33.57	36.06	30.12	34.99	38.67
HepG2	22.85	25.21	25.27	23.10	26.24	25.38
Caco-2	18.98	21.99	20.41	19.25	22.58	21.19

“-“ indicates IC_50_ could not be estimated accurately.

**Table 6:** Apoptotic indices of free 5-FU and 5-FU loaded nanocomposites.

Cell Lines	Apoptosis Index		
	PACTF	CTF	5-FU
HEK293	0.034	0.052	0.389
MCF-7	0.345	0.321	0.361
HepG2	0.542	0.512	0.549
Caco-2	0.621	0.549	0.624

5-FU, 5-Fluorouracil; CTF, CS-TPP-5-FU/Tween 80; PACTF, PtAu-CS-TPP-5-FU/Tween 80.

## Supplementary Materials

The following are available online at https://www.mdpi.com/1999-4923/11/9/439/s1. Table S1: Pharmacokinetic parameters of PACTF under stimulated conditions. Table S2: Pharmacokinetic parameters of CTF under stimulated conditions.
