# Supplementary material for: The Synergism of Platinum-Gold Bimetallic Nanoconjugates Enhances 5-Fluorouracil Delivery In Vitro
Source: Pharmaceutics. 2019 Sep 1;11(9):439. doi: 10.3390/pharmaceutics11090439 (PMC6781313; doi:10.3390/pharmaceutics11090439)
Supplement: Supplementary file 1 [file pharmaceutics-11-00439-s001.pdf]

pharmaceutics

Supplementary Materials: The Synergism of Platinum-Gold Bimetallic Nanoconjugates Enhances 5-Fluorouracil Delivery in Vitro

Vareessh Maney and Moganavelli Singh

Table S1. Pharmacokinetic parameters of PACTF under stimulated conditions.

Environment	Zero-order		First-order		Higuchi		Korsmeyer–Peppas		
	r^2	K_0	r^2	K_1	r^2	K_H	r^2	K_k	n
pH 7.4	0.998	1.598	0.956	0.019	0.986	6.539	0.964	3.847	0.701
pH 6.5	0.998	3.028	0.997	0.044	0.993	12.183	0.924	4.388	0.877
pH 5.0	0.997	3.455	0.990	0.054	0.985	13.908	0.934	6.545	0.789
pH 4.5	0.997	4.077	0.966	0.071	0.959	16.709	0.966	7.486	0.806

K_0, K_1, K_H, and K_k are release rate constants; r^2 is the correlation coefficient; and n is release exponent (indicative of drug release mechanism).

Table S2. Pharmacokinetic parameters of CTF under stimulated conditions.

Environment	Zero-order		First-order		Higuchi		Korsmeyer–Peppas		
	r^2	K_0	r^2	K_1	r^2	K_H	r^2	K_k	n
pH 7.4	0.998	1.917	0.908	0.023	0.945	7.871	0.953	4.175	0.741
pH 6.5	0.998	3.818	0.899	0.062	0.916	15.823	0.965	9.637	0.693
pH 5.0	0.996	4.133	0.923	0.072	0.925	17.101	0.966	10.298	0.697
pH 4.5	0.996	4.457	0.913	0.0838	0.901	18.507	0.963	11.193	0.697

K_0, K_1, K_H, and K_k are release rate constants; r^2 is the correlation coefficient; and n is release exponent (indicative of drug release mechanism).
